# Clinical phenotype and genetic analysis of patients with severe oligoasthenospermia carrying heterozygous SOHLH1 c.346-1G>A mutation

**DOI:** 10.3389/fgene.2025.1531697

**Published:** 2025-01-30

**Authors:** Xiaojun Wen, Zhiming Li, Lizi Cheng, Jianhong Wei, Wenjuan Yu, Xiufeng Lin, Xiaowu Fang

**Affiliations:** ^1^ Reproductive Medicine Center, Boai Hospital of Zhongshan, Zhongshan, Guangdong, China; ^2^ The Second School of Clinical Medicine, Southern Medical University, Guangzhou, Guangdong, China

**Keywords:** male infertility, SOA, SOHLH1 gene, novel clinical phenotype, intracytoplasmic sperm injection

## Abstract

**Introduction:**

Severe oligoasthenospermia (SOA) is a prevalent cause of male infertility. However, the underlying causes of most SOA cases remain unclear due to the complexity of germ cell development and the significant genetic heterogeneity associated with male infertility. Therefore, in this study, we aimed to elucidate the genetic etiology of two cases of male infertility resulting from SOA and clarify the novel clinical phenotype associated with a heterozygous mutation at the c.346-1G>A site of the SOHLH1 gene.

**Methods and results:**

Through whole-exome sequencing, we found that patients with SOA carried heterozygous mutations at the c.346-1G>A site. This variant is classified as pathogenic based on disease database records and literature reports. Notably, our study demonstrated that patients with heterozygous mutations at the c.346-1G>A site exhibited severely reduced sperm counts, significantly impaired sperm motility, and pronounced morphological deformities. One patient underwent assisted reproductive treatment through an intracytoplasmic sperm injection and achieved a favorable outcome, resulting in a successful pregnancy.

**Discussion:**

In conclusion, our study provides the first evidence that the heterozygous mutation at the c.346-1G>A site of SOHLH1 is associated with SOA, and elucidates the new clinical phenotype associated with this mutation.

## Introduction

Globally, approximately 15% of couples experience infertility, with male factors contributing to approximately 50% of cases (Krausz and Riera-Escamilla, 2018). Male infertility is primarily attributed to spermatogenesis disorders, a complex process involving spermatogonial mitosis, spermatocyte meiosis, and transformation of sperm cells into tadpole-shaped sperm. Any abnormalities in these stages can result in male infertility (Neto et al., 2016). Clinical manifestations of spermatogenesis disorders include azoospermia, oligozoospermia, teratozoospermia, and asthenozoospermia, with the most severe cases being azoospermia and severe oligoasthenospermia (SOA), which affect approximately 1% of the global male population (Agarwal et al., 2015). SOA is characterized by an ejaculate sperm content of <5 million/mL (Cooper et al., 2010), reduced sperm motility, forward motility rate <32%, and a percentage of normal morphological sperm lower than 4% of the reference value.

Factors that contribute to SOA primarily involve environmental and genetic factors. Studies indicate that approximately 50% of patients with SOA exhibit some form of genetic abnormality, which can range from variations in chromosome structure or quantity, such as Y chromosome microdeletions, sex chromosome abnormalities (47, XXY), and single gene mutations (Liu et al., 2019; Schlegel et al., 2021; Dohle et al., 2005; Capalbo et al., 2021). However, owing to the intricate and precise process of germ cell development, coupled with the extensive genetic heterogeneity of male infertility, only a small percentage of male patients with infertility receive a definitive genetic diagnosis. Consequently, despite this, the molecular underpinnings of SOA remain poorly understood, with the cause remaining unidentified in approximately 72% of cases (Cooper et al., 2010; Choi et al., 2008). Therefore, in this study, we used whole-exome sequencing to investigate the genetic factors associated with SOA. We identified a heterozygous mutation (c.346-1G>A) in the SOHLH1 gene in two patients with SOA. Notably, these findings suggest a reevaluation of the impact of the c.346-1G>A mutation in the SOHLH1 gene on male infertility, as patients with this mutation exhibit diverse phenotypes that can manifest as azoospermia, normal sperm counts, or severe sperm malformations.

SOHLH1, a germ cell-specific transcription factor, is essential for sperm and egg development. The protein encoded by SOHLH1, which belongs to the basic helix-loop-helix (bHLH) family of transcription factors, contains a helix-loop-helix domain that binds to DNA and facilitates the meiosis process of spermatogenesis (Ballow et al., 2006). In spermatogonial stem cells, SOHLH1 modulates expression of downstream genes by binding to specific DNA sequences (E-box sites) (Tuck et al., 2015). Furthermore, in conjunction with the bHLH transcription factor SOHLH2, SOHLH1 regulates spermatogonial stem cell differentiation by controlling the Kit signaling pathway. SOHLH1 and SOHLH2 can form heterodimers and act on gene promoters, either together or individually, to finely tune spermatogenesis. Studies in mouse models have demonstrated that knockout of SOHLH1 leads to abnormal differentiation of spermatogonia in male mice, resulting in male infertility (Li et al., 2019; Anderson et al., 2008). In previous studies, Choi et al. and Nakamura et al. identified a heterozygous mutation (c.346-1G>A) in SOHLH1 in patients with non-obstructive azoospermia, suggesting a link between heterozygous mutations in this gene and the condition (Choi et al., 2010; Nakamura et al., 2017). Another recent study by Liu et al. demonstrated that the c.346-1G>A heterozygous mutation in SOHLH1 can lead to sperm abnormalities without affecting the sperm count. However, homozygous mutations result in a significant decrease in germ cells and sperm abnormalities (Liu et al., 2022). In this study, we established a new genotype-phenotype association, indicating that the SOHLH1 c.346-1G>A heterozygous mutation is implicated in patients with SOA.

## Materials and methods

### Study participants and ethics approval

First, a comprehensive andrological examination was conducted on patients with SOA, including semen analysis, medical history review, physical examination, hormone analysis, karyotype analysis, and Y chromosome microdeletion screening (AZFa region: sY82, sY84, sY86, sY88, sY1064, and sY1065; AZFb region: sY105, sY121, sY127, sY134, sY153, and sY1192; and AZFc region: sY254 and sY255). Sperm phenotypic abnormalities (severe oligozoospermia, asthenozoospermia, and sperm abnormalities) were diagnosed at least three times, and endocrine abnormalities (hypogonadism, diabetes, and hypothyroidism) and cryptorchidism were absent (Table 1). A two-step analysis was conducted, with the first stage involving whole-exome sequencing of patients with SOA to identify pathogenic factors. In the second stage, patients with heterozygous SOHLH mutations received intracytoplasmic sperm injections (ICSI) to assess their prognosis. This study was approved by the Reproductive Medicine Ethics Committee of Zhongshan Boai Hospital,and the ethical approval number is KY-2020-012-14.

**TABLE 1:** Semen analysis of the spermatozoa.

Parameters	Patient A	Patient B	Control (range)
Sperm concentration (Million/ML)	3.1	0.8	≥15
Total sperm count (Million/one time)	9.8	5.3	≥39
Total sperm motility	3.3	0	≥40
Progressive moeilsty (PR) (%)	0	0	≥32
Non-pcogressive motility (NP) (%)	3.3	0	—
lmmotility (lM) (%)	96.7	100	—
Percentage of normal sperm	0	0	≥4.0%
Sperm count of normal morphology (Normal.)	0	0	—
Number of defective sperm in the head (Head.)	38	70	—
Number of defective sperm in the neck and midsection (Midpiece)	12	34	—
Number of tail defective sperm (Tail.)	8	40	—

### Semen analysis

For semen analysis, patients were instructed to abstain from sex for 3–7 days. Semen was collected via masturbation in a sterile, dry, disposable plastic specimen cup and placed in a 37°C incubator. Once the specimen was completely liquefied, the analysis was completed within 1 h. Following the guidelines outlined in the fifth edition of the ‘World Health Organization Laboratory Manual for the Testing and Processing of Human Semen,’ the appearance, volume, viscosity, liquefaction time, and pH value of the semen were measured after liquefaction (Cooper et al., 2010). Following this, a 3 μL mixed semen sample was analyzed for total sperm count, concentration, and total motility rate using a computer-assisted sperm analysis system (SAS) and SASII version 2.3 software on a disposable sperm counting board. This analysis included progressive motility (PR), non-progressive motility (NP), and sperm morphology. For the latter, a 5 μL completely liquefied semen specimen was dropped on a glass slide, and a semen smear was created using thinning technology. After air-drying and fixing, modified Pap staining was performed to detect sperm morphology. The stained sperm were then analyzed under a light microscope, and over 200 sperm per sample were counted and analyzed to calculate the percentage of sperm with normal morphology.

### Detection of the pathogenic mutation and mutation analysis of the family

Genomic DNA from patients and their parents was extracted from peripheral blood samples using the Qiagen QIAamp DNA Blood Maxi kit (Qiagen, Hilden, Germany) following the manufacturer’s instructions. The DNA quantity and purity were assessed using a Qubit^®^ 3.0 Fluorometer (Thermo Fisher Scientific, MA, United States) and agarose gel electrophoresis to ensure high-quality DNA for further analysis.

Whole-exome sequencing was performed according to the manufacturer’s instructions for library construction and enrichment. Initially, the patient’s DNA was fragmented, and a library was prepared. Subsequently, DNA from the target gene exons and adjacent spliced regions was captured and enriched using the Roche KAPA HyperExome chip. Finally, variant detection was performed using the MGISEQ-2000 sequencing platform. The quality control criteria for sequencing data included an average sequencing depth of the target region ≥180X and a proportion of sites with an average depth of the target region >20X exceeding 95%.

Sequencing fragments were aligned to the UCSC hg19 human reference genome (GRch37: https://www.ncbi.nlm.nih.gov/assembly/GCF_000001405.13/), using BWA for quality control and adapter trimming. Variant sites were identified using GATK HaplotypeCaller, and ANNOVAR (2016Feb01 version) was used for annotation to identify SNV and Indel mutations across the patient’s entire exome. Mutations with allele population frequencies >5% were filtered using databases, such as the 1000 Genomes Project Population Database (http://browser. 1000genomes.org), dbSNP (http://www.ncbi.nlm.nih.gov/snp), and ExAC (http://exac.broadinstitute.org/). The harmful effects of these mutations were predicted using various online prediction software packages (MutationTaster, SIFT, Polyphen-2, and NNSPLICE). Therefore, relevant literature reports from ClinVar (https://www.ncbi.nlm.nih.gov/clinvar/), the human gene mutation database (HGMD^®^) (http://www.hgmd.org), and PubMed were searched to determine the nature of the mutation. The pathogenicity of the mutation was comprehensively assessed based on the pathogenic variant grading guidelines established by the American College of Medical Genetics and Genomics in 2015 (Richards et al., 2015).

Subsequently, the candidate pathogenic loci of the patients and their parents were confirmed using Sanger sequencing. Primers for Sanger sequencing were designed using Oligo 6.0 (http://www.oligo.net/downloads.html), and F: 5′- TGT​GTG​GGG​AAT​GAA​ACT​GT -3′ and R:5′- CCT​GCG​GAG​GCC​AAG​CCG​GG -3 were used to amplify the c.346-1G>A site of SOHLH1 gene.

### Patients with SOHLH heterozygous mutations underwent assisted reproductive therapy with ICSI

Patient A received ICSI treatment using a long luteal phase stimulation protocol. Notably, 1.0 mg of a gonadotropin-releasing hormone an agonist (Difelin, Ipsen) was administered on the 20th day of the menstrual cycle. After 14 days of adjustment, when luteinizing hormone, follicle-stimulating hormone (FSH), estradiol, and progesterone levels met the standard criteria, daily injections of 225 IU gonadotropin (recombinant human FSH, Merck Serono, Switzerland) were initiated. On the 14th day of ovarian stimulation, when at least two follicles reached ≥18 mm in diameter, patients received a single injection of Human Chorionic Gonadotropin (HCG, Merck Serrano, Switzerland) 250 μg to promote the final maturation of follicles and ovulation. Egg retrieval was performed 36 h after HCG injection through a transvaginal ultrasound-guided extraction. On the day of egg retrieval, the male partner provided sperm samples through masturbation. Samples were optimized using density gradient centrifugation and the upstream method before receiving the ICSI.

Notably, all metaphase II (MII) oocytes were subjected to ICSI for fertilization. Early embryo development was monitored at specific time intervals and evaluated based on the early embryo scoring criteria established by Puissant et al. (1987). D3 embryos were transferred to the blastocyst culture medium (Cook or G2 PLUS, Vitrolife, Sweden) and cultured until days 5 or 6. The embryos were subsequently frozen after assessment using blastocyst scoring standards (Gardner et al., 2000). Endometrial preparation involved hormone replacement therapy, and the highest-rated blastocysts were thawed and transferred to the uterus. Blood samples were tested for HCG levels 14 days post-transfer, and intrauterine pregnancy was confirmed using a B-ultrasound 4 weeks post-transfer.

## Results

### Identified heterozygous c.346-1G>A mutation of SOHLH1 in patients with SOA

**FIGURE 1:**
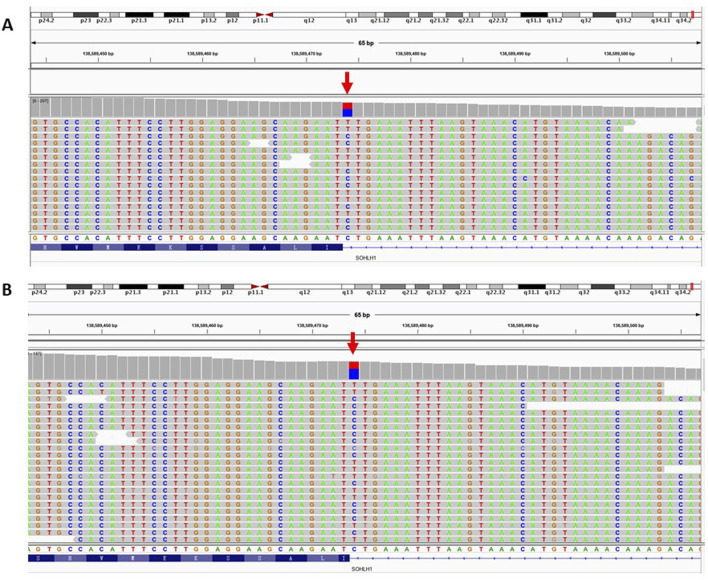
IGV software was used to visualize the patient’s WES results. Figure **(A)** represents the WES result of patient A. Figure **(B)** represents the WES result of patient B. The red arrow represents the SOHLH1 c.346-1G>A site.

**FIGURE 2:**
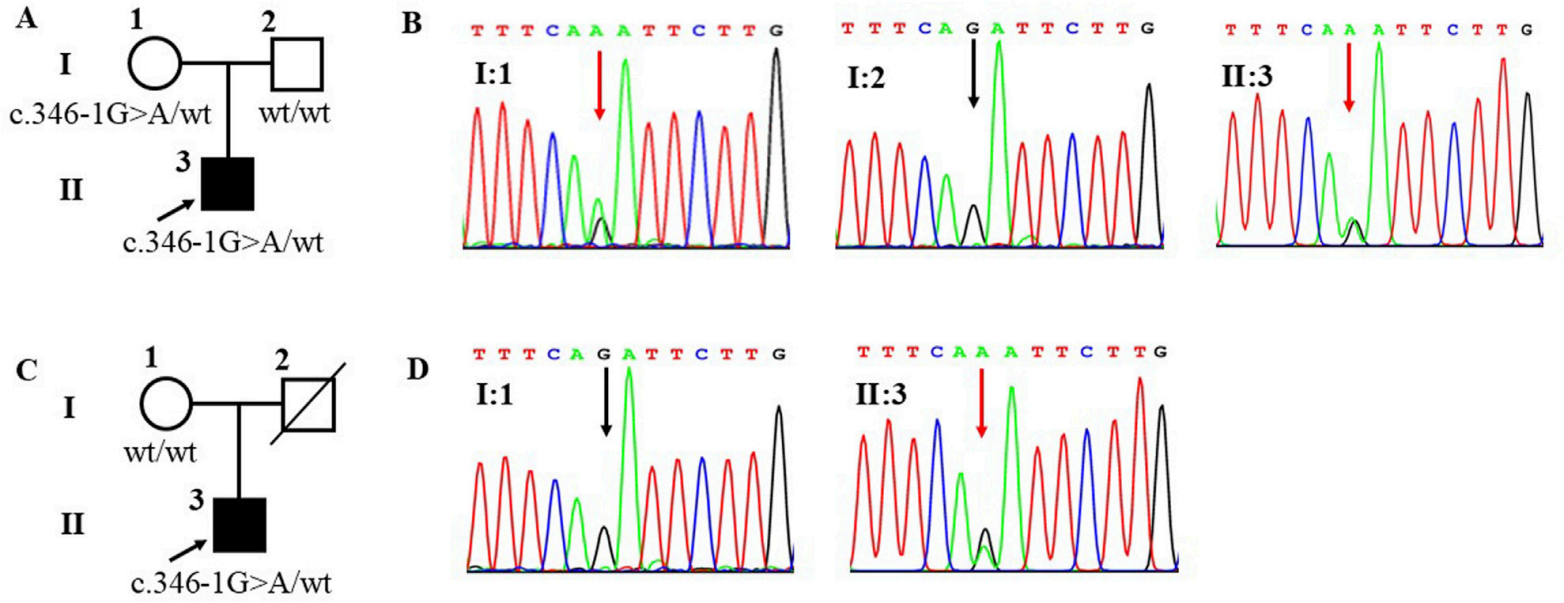
Family pedigrees and Sanger sequencing results of patient A and patient B. Figure **(A)** represents the family pedigree of patient A. Figure **(B)** represents Sanger sequencing results of patient A’s family, the mutation (c.346-1G>A) was observed in I:1 and II:1 but not in I:2. Figure **(C)** represents the family pedigree of patient B. Figure **(D)** represents Sanger sequencing results of patient B’s family, the mutation (c.346-1G>A) was observed in II:3 but not in I:1. A square represents males and a circle represents females. The black filled symbol represents the affected individual and the arrow points to the proband. The slash represents that no sample was collected. The wt represents wildtype. In the Sanger sequencing diagram, the red arrow points to the mutant position and the black arrow points to the reference position.

WES was employed to investigate the genetic etiology of the two patients. A total of 17,223.46 Mb of data was generated, achieving a target area coverage of 99.99% and an average sequencing depth of 249.87X. Following data filtering and analysis, no pathogenic variants associated with SOA were identified, except for the splicing mutation c.346-1G>A in the SOHLH1 gene. The presence of SOHLH1 c.346-1G>A mutation was confirmed using IGV visualization software (Figure 1). According to the HGMD disease database and literature reports (Choi et al., 2010; Nakamura et al., 2017; Liu et al., 2022), the c.346-1G>A variant of SOHLH1 is pathogenic. We performed Sanger sequencing of samples from patients with SOA and their parents to determine the distribution of this mutation (Figure 2). The results indicated that in Patient A’s family, the c.346-1G>A heterozygous mutation in SOHLH1 was inherited from his mother, whereas his father did not carry this mutation. In Patient B’s family, samples could not be obtained for verification because the patient’s father had passed, and his mother did not carry the mutation.

### Clinical phenotypes of patients

According to the routine semen analysis results (Table 1), the sperm concentrations for Patients A and B were 3.1 × 10^6^ mL^−1^ and 0.8 × 10^6^ mL^−1^, respectively, with total sperm counts of 9.8 million and 5.3 million per specimen. The total sperm motility, progressive motility, non-progressive motility, and immotile sperm rates in Patient A were 3.3%, 0%, 3.3%, and 96.7%, respectively. However, Patient B exhibited a total motility of 0%, indicating that all sperm were immotile. The modified Papanicolaou staining method was used to assess sperm morphology, revealing that Patients A and B had sperm with abnormal morphology. These semen analysis results suggested that the c.346-1G>A heterozygous mutation in SOHLH1 may be responsible for the severe reduction in sperm count, decreased sperm motility, and sperm deformities observed. Furthermore, sex hormone test results indicated that Patient A’s hormone levels were within the normal range, whereas Patient B’s prolactin level was elevated, which may be a physiological response. However, the Y chromosome microdeletion and karyotype analyses for Patients A and B returned normal results (Table 2).

**TABLE 2:** Summary of the clinical features of two patients.

Parameters	Patient A	Patient B	Control (range)
Size of both testicles (mL)	16	20	15–25
Human follicle stimulating hormone (FSH) (mIU/ml)	5.44	1.6	1.27–19.3
Human luteinizing hormone (LH) (mIU/ml)	3.3	2.17	1.24–8.62
Prolactin (PRL) (ng/ml)	9.23	17.59	2.6–13.1
Testosterone(T) (ng/ml)	5.71	2.92	1.75–7.81
estradiol (E2) (g/ml)	18.34	35.32	15–38.95
Ychromosome microdeletion screening	Normal	Normal	—
Chromosome karyotype	46,XY	46,XY	—

### Results of ICSI-assisted pregnancy therapy in patients with SOHLH1 gene c.346-1G >A mutation

Patient A underwent ICSI-assisted reproductive treatment after providing informed consent. Following a thorough examination, the basal hormone levels of the patient’s wife were normal (Table 3). Under a long stimulation protocol, 14 mature oocytes (MII) were collected for ICSI fertilization. Of these, 10 oocytes were successfully fertilized, resulting in the formation of a two-pronuclear zygote. After embryo culture, 10 embryos were obtained on day 3, six of which were classified as high-quality embryos (9I, 9II, 8II, 8II, 8II, 8II). Following sequential culture, four blastocysts were formed (4AA, 4AA, 4AB, and 4BB). Embryos with the highest morphological score (4AA) were prioritized for transfer to the uterus. On the 12th day post-transplantation, the blood HCG value was measured at 204.32 mIU/mL. Furthermore, by the 35th day post-transplantation, the fetal heartbeat and embryo were visible on B-ultrasound, with no significant complications observed; this confirmed a successful pregnancy. This study demonstrates that patients with severe oligozoospermia, asthenozoospermia, and teratozoospermia due to the SOHLH1 c.346-1G>A heterozygous mutation can be effectively treated with ICSI-assisted reproductive techniques, yielding a favorable prognosis.

**TABLE 3:** Clinical features of the patient with ICSI.

Items	Patient A
Male age (years)	30
Female age (years)	30
Length of primary infertility history(y)	3
Basal sexual hormone FSH (IU/L)	7.14
LH(IU/L)	4.98
T (nmol/L)	0.42
E2 (pg/mL)	26.5
AMH(ng/mL)	3.42
Protocol	Long
No. of oocytes retrieved	14
No. of mutured oocytes (MII)	14
No. of normal fertilization (2 PN)	10
Fertilization rate (%)	100 (14/14)
Cleavage rate (%))	71.43 (10/14)
Available D3 embryos	8
Blastocyst formation rate (%)	50.00 (4/8)

## Discussion

Spermatogenesis is a fundamental aspect of the male reproductive system that involves the intricate processes of cell differentiation and development. Previous studies have established that SOHLH1 is a germ cell-specific transcription factor that is critical in the development and differentiation of germ cells. Barrios et al. and Anderson et al. demonstrated that SOHLH1 regulates the transcription of critical genes, such as Kit, Neurogenin 3, stimulated by retinoic acid 8, and Synaptonemal Complex Protein 1/3, during spermatogenesis, thereby influencing sperm development (Anderson et al., 2008; Barrios et al., 2012). Suzuki et al. found that both SOHLH1 and SOHLH2 affect spermatogonial development by directly regulating anti-glial cell line-derived neurotrophic factor family receptor α 1 and SRY-box transcription factor 3 (Suzuki et al., 2012).

In this study, two patients with severe oligozoospermia, asthenozoospermia, and teratozoospermia were found to carry a heterozygous SOHLH1 mutation at the c.346-1G>A site. Notably, multiple reports have indicated that certain mutations in the SOHLH1 gene are associated with non‐obstructive azoospermia (NOA). Choi et al. analyzed 96 Korean men with non-obstructive azoospermia and discovered that twopatients in their study carried a splice-site mutation (c.346-1G>A) in the SOHLH1 gene (Choi et al., 2010). Reverse transcription-polymerase chain reaction analysis revealed that the transcript mutated at this site was shorter than the wild type. Furthermore, sequence analysis confirmed an in-frame deletion of 18 bp in exon 4, resulting in truncation of the bHLH domain. In transcription activity assays, the activity of the mutant protein was less than half that of the wild-type protein, confirming that the heterozygous SOHLH1 mutation at the c.346-1G>A site is implicated in male NOA. Subsequently, in 2017, Nakamura et al. analyzed 25 azoospermia-related genes in 40 Japanese men with NOA and identified the SOHLH1 c.346-1G>A mutation in two patients (Nakamura et al., 2017). In 2022, Liu et al. revised the previous conclusion that the heterozygous mutation c.346-1G>A in SOHLH1 was responsible for azoospermia. In the current study, Liu et al. demonstrated that the homozygous mutation c.346-1G>A in SOHLH1 is the genetic cause of human azoospermia, indicating that defects in spermatogenesis are associated with homozygous rather than heterozygous mutations. Specifically, patients with the heterozygous c.346-1G>A mutation in SOHLH1 exhibit normal sperm counts, whereas those with the homozygous mutation show severely reduced sperm counts (Liu et al., 2022).

In summary, this study revealed that the clinical phenotype of patients carrying the c.346-1G>A heterozygous mutation differs from the findings of previous studies. Notably, both patients exhibited severely reduced sperm count and significant deformities. Patient A had a sperm concentration of 3.1 × 10^6^ mL^−1^ and sperm motility of 3.3%. However, Patient B demonstrated an even more severe clinical phenotype, with a sperm concentration of only 0.8 × 10^6^ mL^−1^ and sperm motility of 0%. Notably, both patients had 0% normal sperm with sperm abnormalities primarily characterized by defects in the sperm head. Additionally, we analyzed the pedigrees of the two patients. The c.346-1G>A heterozygous mutation in Patient A’s SOHLH1 was inherited from his mother. In contrast, Patient B’s mother did not carry this mutation. Consequently, because his father passed away without a sample being collected, it remains unclear whether the mutation was inherited from his father or represents a *de novo* mutation.

Previous studies have indicated that ICSI yields favorable outcomes in men with severe infertility attributable to genetic mutations (Hua et al., 2023). Additionally, studies have reported that mutations in male factor-related genes such as phospholipase C zeta 1, actin-like 7A, and actin-like 9 may contribute to fertilization failure during ICSI (Sha et al., 2022; Xue et al., 2022). However, studies on ICSI treatment in patients with SOHLH1 mutations are lacking. Liu et al. reported two patients with the SOHLH1 c.346-1G>A heterozygous mutation and one patient with the SOHLH1 c.346-1G>A mutation, all of whom underwent ICSI-assisted pregnancy, resulting in successful births (Liu et al., 2022). Patient A also underwent ICSI-assisted reproductive treatment. Following semen processing using the SpermGrad gradient centrifugation method, the sperm viability rate was 90%, with a progressive motility rate of 85%, a non-progressive motility rate of 5%, and an immotile sperm rate of 10%. The embryo fertilization rate reached 100%, and the blastocyst formation rate was 50%. Furthermore, the patient’s wife successfully became pregnant after embryo transfer. Therefore, we demonstrated that despite severely reduced sperm count and motility and significant morphological abnormalities associated with the SOHLH1 c.346-1G>A heterozygous mutation, patients can still achieve favorable outcomes following assisted reproductive procedures utilizing ICSI technology with normal fertilization and embryonic development observed.

This study has some limitations. First, only two patients with a severe reduction in sperm count, low sperm motility, and malformations were identified as carrying the SOHLH1 c.346-1G>A heterozygous mutation, resulting in a small sample size. Second, Patient B, who presented with a more severe clinical phenotype, had not yet undergone ICSI-assisted reproductive treatment, preventing the evaluation of her prognosis. Finally, both patients in this study were of Chinese ethnicity, which may limit the applicability of our findings to other ethnic groups because the clinical phenotypes associated with the SOHLH1 c.346-1G>A mutation may vary across populations.

In conclusion, this study identified the heterozygous mutation SOHLH1 c.346-1G>A in two patients with spermatogenic failure, associating it with reduced sperm count, impaired motility, and morphological abnormalities. Intracytoplasmic sperm injection (ICSI) was successful in one case, highlighting the potential for genetic counseling and assisted reproductive technologies in managing male infertility attributed to SOHLH1 mutations.

## Data Availability

The data presented in the study are deposited in the CNGB Sequence Archive (CNSA) of China National GeneBank DataBase (CNGBdb), accession number CNP0006678, https://db.cngb.org/search/project/CNP0006678/.

## References

[B1] Agarwal A. Mulgund A. Hamada A. Chyatte M. R. (2015). A unique view on male infertility around the globe. Reproductive Biol. Endocrinol. RB&E 13, 37. 10.1186/s12958-015-0032-1 25928197 PMC4424520

[B2] Anderson E. L. Baltus A. E. Roepers-Gajadien H. L. Hassold T. J. de Rooij D. G. van Pelt A. M. (2008). Stra8 and its inducer, retinoic acid, regulate meiotic initiation in both spermatogenesis and oogenesis in mice. Proc. Natl. Acad. Sci. U. S. A. 105, 14976–14980. 10.1073/pnas.0807297105 18799751 PMC2542382

[B3] Ballow D. Meistrich M. L. Matzuk M. Rajkovic A. (2006). Sohlh1 is essential for spermatogonial differentiation. Dev. Biol. 294, 161–167. 10.1016/j.ydbio.2006.02.027 16564520

[B4] Barrios F. Filipponi D. Campolo F. Gori M. Bramucci F. Pellegrini M. (2012). SOHLH1 and SOHLH2 control Kit expression during postnatal male germ cell development. J. Cell. Sci. 125, 1455–1464. 10.1242/jcs.092593 22328502

[B5] Capalbo A. Poli M. Riera-Escamilla A. Shukla V. Kudo Høffding M. Krausz C. (2021). Preconception genome medicine: current state and future perspectives to improve infertility diagnosis and reproductive and health outcomes based on individual genomic data. Hum. Reprod. update 27, 254–279. 10.1093/humupd/dmaa044 33197264

[B6] Choi Y. Jeon S. Choi M. Lee M. H. Park M. Lee D. R. (2010). Mutations in SOHLH1 gene associate with nonobstructive azoospermia. Hum. Mutat. 31, 788–793. 10.1002/humu.21264 20506135

[B7] Choi Y. Yuan D. Rajkovic A. (2008). Germ cell-specific transcriptional regulator sohlh2 is essential for early mouse folliculogenesis and oocyte-specific gene expression. Biol. reproduction 79, 1176–1182. 10.1095/biolreprod.108.071217 PMC2780471 18753606

[B8] Cooper T. G. Noonan E. von Eckardstein S. Auger J. Baker H. W. Behre H. M. (2010). World Health Organization reference values for human semen characteristics. Hum. Reprod. update 16, 231–245. 10.1093/humupd/dmp048 19934213

[B9] Dohle G. R. Colpi G. M. Hargreave T. B. Papp G. K. Jungwirth A. Weidner W. (2005). EAU guidelines on male infertility. Eur. Urol. 48, 703–711. 10.1016/j.eururo.2005.06.002 16005562

[B10] Gardner D. K. Lane M. Stevens J. Schlenker T. Schoolcraft W. B. (2000). Blastocyst score affects implantation and pregnancy outcome: towards a single blastocyst transfer. Fertil. Steril. 73, 1155–1158. 10.1016/s0015-0282(00)00518-5 10856474

[B11] Hua R. Xue R. Liu Y. Li Y. Sha X. Li K. (2023). ACROSIN deficiency causes total fertilization failure in humans by preventing the sperm from penetrating the zona pellucida. Hum. Reprod. Oxf. Engl. 38, 1213–1223. 10.1093/humrep/dead059 37004249

[B12] Krausz C. Riera-Escamilla A. (2018). Genetics of male infertility. Nat. Rev. Urol. 15, 369–384. 10.1038/s41585-018-0003-3 29622783

[B13] Li Y. Qi W. Liu G. Du B. Sun Q. Zhang X. (2019). Sohlh1 is required for synaptonemal complex formation by transcriptionally regulating meiotic genes during spermatogenesis in mice. Mol. reproduction Dev. 86, 252–264. 10.1002/mrd.23100 30614095

[B14] Liu C. He X. Liu W. Yang S. Wang L. Li W. (2019). Bi-Allelic mutations in TTC29 cause male subfertility with asthenoteratospermia in humans and mice. Am. J. Hum. Genet. 105, 1168–1181. 10.1016/j.ajhg.2019.10.010 31735294 PMC6904824

[B15] Liu M. Yang Y. Wang Y. Chen S. Shen Y. (2022). The mutation c.346-1G > A in SOHLH1 impairs sperm production in the homozygous but not in the heterozygous condition. Hum. Mol. Genet. 31, 1013–1021. 10.1093/hmg/ddab242 34448846 PMC8976425

[B16] Nakamura S. Miyado M. Saito K. Katsumi M. Nakamura A. Kobori Y. (2017). Next-generation sequencing for patients with non-obstructive azoospermia: implications for significant roles of monogenic/oligogenic mutations. Andrology 5, 824–831. 10.1111/andr.12378 28718531

[B17] Neto F. T. Bach P. V. Najari B. B. Li P. S. Goldstein M. (2016). Spermatogenesis in humans and its affecting factors. Seminars Cell. and Dev. Biol. 59, 10–26. 10.1016/j.semcdb.2016.04.009 27143445

[B18] Puissant F. Van Rysselberge M. Barlow P. Deweze J. Leroy F. (1987). Embryo scoring as a prognostic tool in IVF treatment. Hum. Reprod. Oxf. Engl. 2, 705–708. 10.1093/oxfordjournals.humrep.a136618 3437050

[B19] Richards S. Aziz N. Bale S. Bick D. Das S. Gastier-Foster J. (2015). Standards and guidelines for the interpretation of sequence variants: a joint consensus recommendation of the American College of medical genetics and Genomics and the association for molecular pathology. official J. Am. Coll. Med. Genet. 17, 405–424. 10.1038/gim.2015.30 PMC4544753 25741868

[B20] Schlegel P. N. Sigman M. Collura B. De Jonge C. J. Eisenberg M. L. Lamb D. J. (2021). Diagnosis and treatment of infertility in men: AUA/ASRM guideline part I. Fertil. Steril. 115, 54–61. 10.1016/j.fertnstert.2020.11.015 33309062

[B21] Sha Y. Chen Y. Wang X. Meng R. Yang X. Li Y. (2022). Biallelic mutations in IQCN, encoding a novel acroplaxome protein, lead to fertilization failure and male infertility with defects in the acrosome and shaping of the spermatid head in humans and mice. Life Med. 2. 10.1093/lifemedi/lnac050 PMC11749140 39872118

[B22] Suzuki H. Ahn H. W. Chu T. Bowden W. Gassei K. Orwig K. (2012). SOHLH1 and SOHLH2 coordinate spermatogonial differentiation. Dev. Biol. 361, 301–312. 10.1016/j.ydbio.2011.10.027 22056784 PMC3249242

[B23] Tuck A. R. Robker R. L. Norman R. J. Tilley W. D. Hickey T. E. (2015). Expression and localisation of c-kit and KITL in the adult human ovary. J. ovarian Res. 8, 31. 10.1186/s13048-015-0159-x 26008799 PMC4460643

[B24] Xue Y. Cheng X. Xiong Y. Li K. (2022). Gene mutations associated with fertilization failure after *in vitro* fertilization/intracytoplasmic sperm injection. Front. Endocrinol. 13, 1086883. 10.3389/fendo.2022.1086883 PMC9800785 36589837

